# Osmotic Demyelination Syndrome Despite Appropriate Hyponatremia Correction

**DOI:** 10.7759/cureus.8209

**Published:** 2020-05-20

**Authors:** Mansura Jahan, Shorabh Sharma, Razia Rehmani

**Affiliations:** 1 Internal Medicine, St. Barnabas Hospital Health System, Bronx, USA; 2 Radiology, St. Barnabas Hospital Health System, Albert Einstein College of Medicine, Bronx, USA

**Keywords:** hyponatremia, invasive colon cancer, alcohol withdrawal, osmotic demyelination syndrome

## Abstract

Acute demyelination of the pons or extrapontine areas results in an osmotic demyelination syndrome (ODS), previously referred to as central pontine myelinolysis (CPM) or extra pontine myelinolysis (EPM). It is caused by osmotic dysregulation in the brain. Multiple risk factors have been known to contribute to these osmotic disturbances. Among them, osmotic stress caused by rapid correction of hyponatremia is the most common cause. Other risk factors include liver failure, alcohol dependence, malnutrition, and malignancy. Symptoms can vary depending on the location of the demyelination. It has a high rate of morbidity and mortality. We present a case of ODS in a malnourished patient who was found to have alcoholic hepatitis and invasive colon cancer. The initial presentation was sepsis secondary to pneumonia. The patient was found to be severely hyponatremic at the time of admission, and the hyponatremia was corrected as per the recommendations. The initial non-contrast head computed tomography (CT) scan was unremarkable. However, the hospital course was complicated by a deteriorating neurological exam with encephalopathy despite not overcorrecting the sodium. A short-term follow-up brain magnetic resonance imaging (MRI) eventually revealed ODS. Initially, the findings of ODS were masked due to symptoms of alcohol withdrawal. However, the patient had a quick recovery with the improvement of all the neurological findings.

## Introduction

Osmotic demyelination syndrome (ODS) is caused by demyelination of the pons or extra pontine areas [1]. Previously this used to be called central pontine myelinolysis (CPM) and extra pontine myelinolysis (EPM). Demyelination in the central nervous system is usually precipitated by the aggressive correction of a hyper or hypo-osmolar condition [2]. Other risk factors that can contribute to ODS are malnutrition, hepatic failure, sepsis, malignancy, chronic renal failure with dialysis, severe burns, advanced lymphoma, carcinoma, cachexia, severe bacterial infections, acute hemorrhagic pancreatitis, pregnancy or postpartum state, adrenal insufficiency, metabolic derangement, and pellagra [1-2]. The brain has few adaptive mechanisms because of hyponatremia. Once the brain adapts to hyponatremia, it is not well-protected from the osmotic stress that occurs during the correction of the hyponatremia [3]. Usually, alcoholics and malnourished patients have a general deficiency of organic osmolytes, which puts them at greater risk of cell shrinkage [4]. CPM involves the basis pontis symmetrically, the pontocerebellar fibers, and usually spares the ventrolateral pons. EPM commonly involves the basal ganglia, thalami, and cerebral white matter [5]. We are presenting the case of an alcoholic patient who developed ODS despite the normal rate of the correction of hyponatremia. We would like to emphasize the fact that multiple risk factors contributed to the development of ODS in this particular case, despite the appropriate correction of sodium. The prognosis of ODS has improved significantly because of an magnetic resonance imaging (MRI)-based early diagnosis. However, 33% to 55% of patients can die or remain in a permanent vegetative state [6]. 

## Case presentation

**Figure 1:**
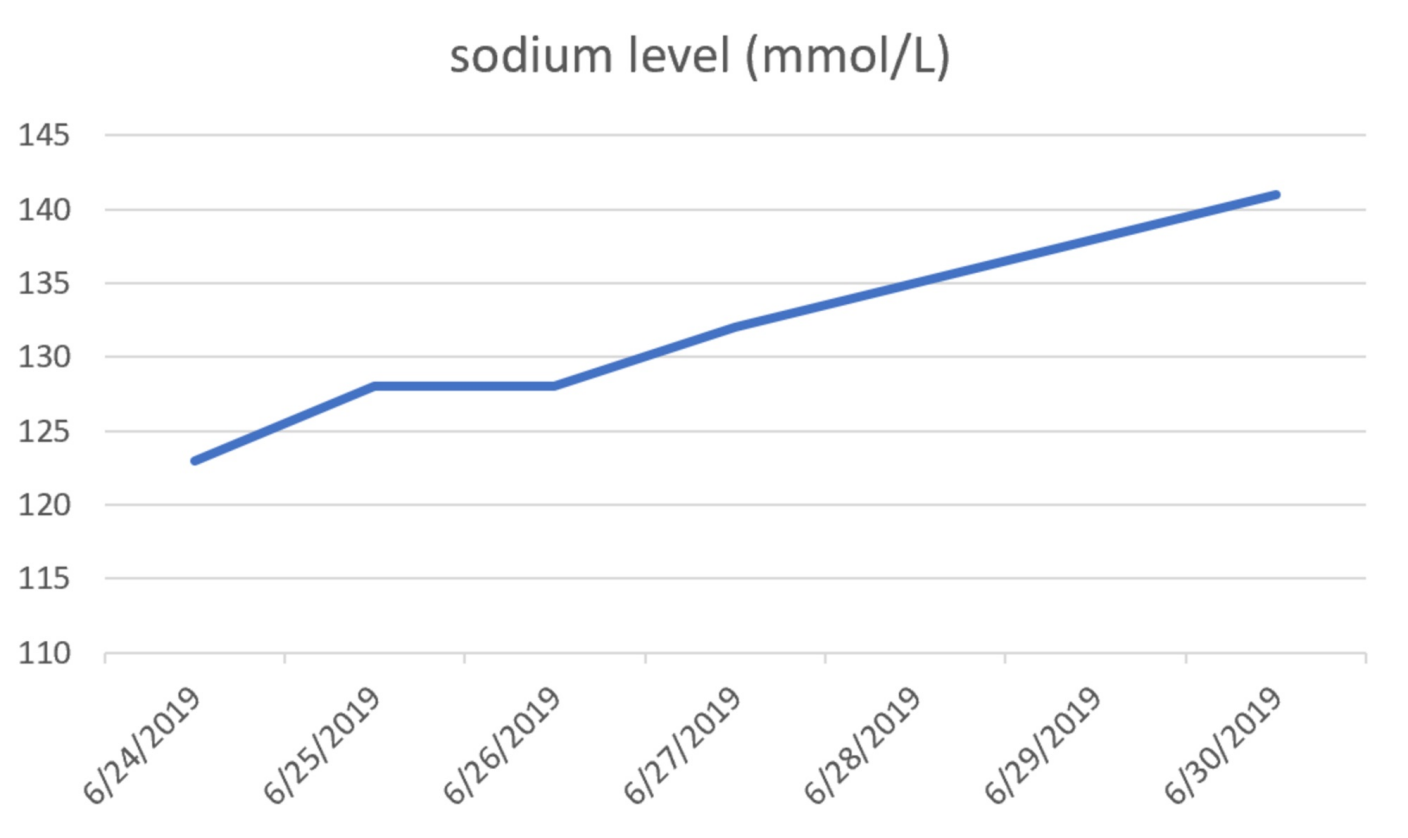
Appropriate correction of sodium at a rate of less than 8 mmol/L/24 hours

A 55-year-old male with a history of alcohol and polysubstance use disorder presented to the emergency department with complaints of progressively worsening leg weakness, frequent falls, unintentional weight loss, and non-bloody diarrhea over a period of two weeks. Initial blood chemistry showed hyponatremia with a sodium level of 123 mEq/L (normal: 135 to 145 mEq/L). Random urine sodium was 26 mEq/L (normal: 20 mEq/L in a random urine sample and 40 to 220 mEq/L per day). The patient's sodium was corrected with free water restriction and gentle intravenous hydration with normal saline (Table 1). Figure 1 below shows the appropriate correction of sodium at a rate of less than 8 mmol/L/24 hours. The following week, the patient was found to be encephalopathic with symptoms of drowsiness, dysarthria, dysphagia, and ophthalmoplegia with an increased tone in both upper and lower extremities. On examination, deep tendon reflexes were increased equally; Babinski sign was negative without any clonus.

**Table 1:** The Rate of Correction of Hyponatremia Per Day Over a Five Day Period

Date	Rate of sodium correction in 24 hours
6/24/2019	0
6/25/2019	5
6/26/2019	0
6/27/2019	4
6/30/2019	9

**Figure 2:**
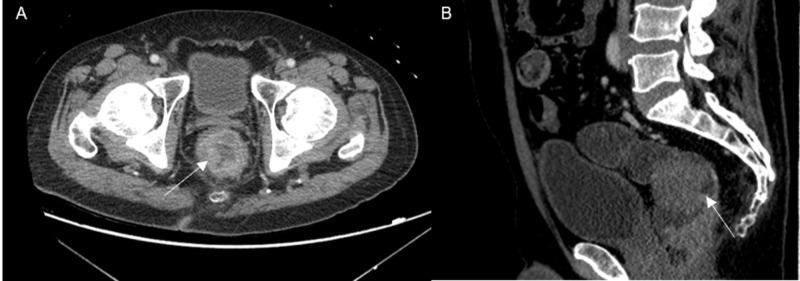
Contrast-enhanced computed tomography (CT) scan of the pelvis Axial (A) and sagittal (B) images from contrast-enhanced CT of the pelvis showed a large rectosigmoid mass (arrow) suggestive of a colorectal malignancy

**Figure 3:**
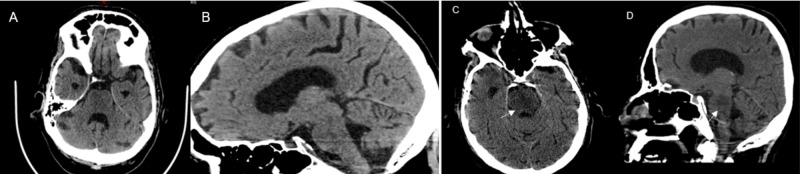
Initial and repeat non-contrast computed tomography (CT) scans of the brain (A) Axial and (B) sagittal views on a non-contrast CT head scan showing no abnormalities; (C) axial and (D) sagittal views on a non-contrast CT head scan three weeks later showing hypodensity (arrow) in the central pons, suggesting central pontine myelinolysis (CPM)

**Figure 4:**
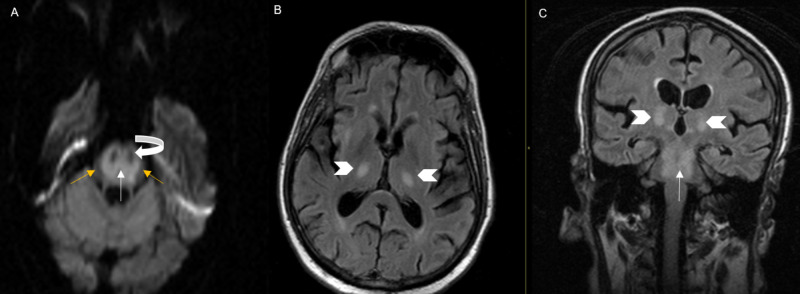
Axial diffusion-weighted (DWI) magnetic resonance imaging (MRI) (A) MRI demonstrates diffusion restriction involving the central pons (white arrow), sparing the periphery (yellow arrow), as well as corticospinal tract (curved white arrow). (B) Axial and (C) coronal flair images showing extra pontine involvement with T2 prolongation involving the bilateral pons (arrowhead) in addition to the central pons (white arrow).

Computed tomography (CT) scan of the abdomen and pelvis demonstrated a rectosigmoid mass (Figure 2). Initial head CT was unremarkable for acute intracranial pathology (Figure 3A-B). However, repeat CT brain was (Figure 3C-D) done that demonstrated hypodensity suggestive of ODS. MRI scan of the brain was done that also revealed diffusion restriction suggestive of pontine and extra pontine myelinolysis (Figure 4).

Following this, supportive treatment was offered. Over time, the patient’s dysarthria, dysphagia, and neurological examination improved significantly. The patient underwent a colonoscopy and biopsy of the lesion which showed invasive colon adenocarcinoma. The patient completed the treatment with surgical resection and chemotherapy. Malignancy can also be a contributing factor to ODS, as in our case. Further research needs to complete to understand the pathophysiology of cancer and chemotherapy as the cause of ODS.

## Discussion

ODS is usually accelerated by the aggressive correction of a hyper or hypo-osmolar condition [2]. When a patient has hyponatremia, sodium and potassium concentrations in the brain decrease. The brain also loses different anions. With these adaptive mechanisms, the water content in the brain also returns to normal within 48 hours. Once the brain adapts to hyponatremia, it is not well-protected from the osmotic stress that occurs during the correction of hyponatremia [3]. If there is rapid correction of a chronic osmolar abnormality when there is already a deficit of organic osmolytes, it places brain cells, particularly oligodendrocytes, at risk of cell shrinkage and can lead to demyelination. Usually, alcoholics and malnourished patients have a general deficiency of organic osmolytes, which puts them at greater risk of cell shrinkage [4]. It may consist of CPM and/or EPM. CPM involves the basis pontis symmetrically, pontocerebellar fibers, and usually spares the ventrolateral pons. EPM commonly involves the basal ganglia, thalami, and cerebral white matter [5]. MRI of the brain is the best diagnostic modality. The prognosis has improved significantly as a result of an MRI-based early diagnosis of CPM. However, it still can be fatal and 33% to 55% of patients can die or remain in a permanent vegetative state [6]. Among variable risk factors, chronic alcoholism is considered as the most common cause of ODS [7-8]. The incidence of ODS is approximately 39.4% in alcoholics, followed by 21.5% due to rapid correction of hyponatremia, and 17.4% in liver transplant patients, which is being attributed to immunosuppressive agents, like cyclosporine [8]. Other risks factors that can contribute ODS are malnutrition, hepatic failure, sepsis, malignancy, chronic renal failure with dialysis, severe burns, advanced lymphoma, carcinoma, cachexia, severe bacterial infections, acute hemorrhagic pancreatitis, pregnancy or postpartum state, adrenal insufficiency, metabolic derangement, and pellagra [1-2, 9]. 

The clinical presentations may vary depending on the degree of pontine involvement and the presence of extrapontine lesions. The patient can be asymptomatic or present with progressive lethargy, quadriparesis, dysarthria, ophthalmoplegia, and ataxia [9-10]. In some cases, it can lead to coma or death [11]. Sometimes the symptoms of ODS can be similar to those seen in alcohol withdrawal. Therefore, early recognition of neurological changes due to the development of ODS in an alcoholic patient can be challenging. A prolonged encephalopathy beyond the expected duration of delirium tremens (one to five days) should raise concern for ODS. A high degree of clinical suspicion is required to diagnose CPM [9]. It has been described previously that chronic alcoholism-associated ODS has a favorable prognosis compared to ODS secondary to the rapid correction of hyponatremia [9]. For hyponatremic patients, the recommended correction rate is not more than > 0.5 mmol/L/hr, with a maximum of 12 mmol/L over 24 hours and 25 mmol/L over 48 hours. It is suggested to lower rates in higher-risk individuals, with a goal of 8 mmol/L in the first 24 hours [1, 12].

Early diagnosis of ODS is challenging as it may not show up on CT in the initial encephalopathy stages [2, 13]. However, sometimes, a CT scan can detect low-attenuation changes in the pons. An MRI scan of the brain is more sensitive for detecting an increase in the tissue water content and demyelination on fluid-sensitive sequences [2, 14-16]. MRI findings include symmetric hypointensity on T1-weighted images, hyperintensity on T2-weighted, and fluid-attenuated inversion recovery (FLAIR) images with or without diffusion restriction. It commonly involves the basis pontis and extends from the pontomedullary junction into the midbrain with characteristic sparing of the tegmentum. In more severe disease, almost all of the entire central pons may be involved with only a thin rim of normal signal around it [2, 17]. New lesions are symmetrical and hypointense on T1-weighted images in the acute stage, while subacute cases are hyperintense on T2-weighted images. In the early phase of the disease, the findings may not be clearly visible on the images as the demyelinated patches do not show up until one to two weeks later. Repeat imaging after 10 to 14 days is recommended [8]. In certain cases where CT or MRI are inconspicuous, diffusion-weighted imaging (DWI) is useful in the early diagnosis of CPM [11]. In the acute phase, restricted diffusion is shown on DWI with corresponding low apparent diffusion coefficient (ADC) values. This appearance on DWI and the ADC values suggest the presence of cytotoxic edema in acute CMP. Here, the MRI finding showed ODS in our patient. A follow-up examination may show normalization of the DWI signal and ADC values, suggesting the disappearance of cytotoxic edema in the later phase [2, 17]. There is no therapy of choice for the management of ODS. Administration of thyrotropin-releasing hormone (TRH), plasmapheresis, and corticosteroids alone or in combination with plasmapheresis, as well as intravenous immunoglobulins, may have a better outcome [8].

## Conclusions

ODS is caused by osmotic disturbances leading to a demyelinating injury which is commonly believed to be caused by the rapid correction of hyponatremia. Multiple additional risk factors were identified in our case, in addition to hyponatremia, inclusive of chronic alcoholic hepatitis, sepsis, and gastrointestinal malignancy. In a patient with chronic hyponatremia, it is imperative to correct sodium more conservatively. Other causes of encephalopathy, such as ischemia, hypoxia, or toxicity, also need to be excluded before diagnosing ODS. The clinician needs a high level of suspicion to detect ODS as early changes may be obscured on conventional CT. Furthermore, diagnosing ODS in alcoholic patients is challenging as symptoms of ODS can overlap with alcohol withdrawal. In our patient, the diagnosis of ODS was masked by alcohol withdrawal. However, the patient recovered quickly without any residual symptoms. Clinicians also need to be aware of malignancy during the diagnosis and treatment as it can be linked with ODS. Further research needs to be completed to understand the pathophysiology of cancer and chemotherapy as the cause of ODS.
